# Assessment of the Impact of the Application of a Quercetin—Copper Complex on the Course of Physiological and Biochemical Processes in Wheat Plants (*Triticum aestivum* L.) Growing under Saline Conditions

**DOI:** 10.3390/cells11071141

**Published:** 2022-03-28

**Authors:** Marta Jańczak-Pieniążek, Dagmara Migut, Tomasz Piechowiak, Maciej Balawejder

**Affiliations:** 1 Department of Crop Production, University of Rzeszow, Zelwerowicza 4, 35-601 Rzeszow, Poland; dmigut@ur.edu.pl; 2 Department of Food Chemistry and Toxicology, University of Rzeszow, Ćwiklińskiej 1A, 35-601 Rzeszów, Poland; tpiechowiak@ur.edu.pl (T.P.); maciejb@ur.edu.pl (M.B.)

**Keywords:** wheat, salt stress, quercetin derivative, Cu(II)–quercetin complex, reactive oxygen species, antioxidant enzymes, chlorophyll content, chlorophyll fluorescence, gas exchange

## Abstract

Salt stress is one of the main stressors limiting plant growth and yield. As a result of salt stress, unfavorable changes in the photosynthesis process take place, leading to a decrease in plant productivity. Therefore, it is necessary to use biologically active substances that reduce the effects of this stress. An example of such a substance is quercetin, classified as a flavonoid, which plays an important role in alleviating the effects of salt stress, mainly by the inactivation of reactive oxygen species (ROS) and by improvement of the photosynthesis process. A study was made of the effect of the quercetin–copper complex (Q-Cu (II)), which has a stronger antioxidant effect than pure quercetin. By means of a pot experiment, the influence of solutions of the Q-Cu (II) complex (100 mg∙L^−1^ [Q1], 500 mg∙L^−1^ [Q2] and 1000 mg∙L^−1^ [Q3]) on the physiological and biochemical processes occurring in wheat plants subjected to salt stress was investigated. The plants were given two sprays of Q-Cu (II) solution, and their physiological parameters were examined both 1 and 7 days after each application of this solution. The level of ROS and the activity of antioxidant enzymes (catalase [CAT], superoxide dismutase [SOD] and guaiacol peroxidase [GPOX]) were also determined. It has been shown that spraying with Q2 and Q3 solutions improves the chlorophyll content, the values of chlorophyll fluorescence parameters (the photochemical efficiency of PS II [F_v_/F_m_], the maximum quantum yield of primary photochemistry [F_v_/F_0_], and the performance index of PS II [PI]), and gas exchange (net photosynthetic rate [P_n_], stomatal conductance [g_s_], transpiration rate [E] and intercellular CO_2_ concentration [C_i_]). As a result of the application of Q2 and Q3 solutions, the level of ROS and the activity of the antioxidant enzymes tested decreased, which means that these concentrations are most effective in counteracting the effects of salt stress.

## 1. Introduction

Wheat (*Triticum aestivum* L.) is the dominant species in cultivation in regions with a temperate climate due to its wide use as human food and animal feed [1,2]. The great importance of wheat results from the content of bioactive substances in the grain that have a positive effect on human health [3]. Therefore, it plays a significant role in the human diet, and the resulting flour is the raw material for the production of many products that people consume, including bread [4]. The total area under cultivation in the world is 219 million ha, while the yield is 3.5 t∙ha^−1^ [5]. During cultivation, wheat is exposed to various environmental stresses, including soil salinity [6]. The problem of soil salinity is of especial note in arid and semi-arid regions. At present, it is estimated that this problem affects about 6% of the total land area, 22% of which is arable land. Soil salinity is largely associated with activities related to agricultural practices (soil irrigation, fertiliser use) [7], forming one of the main threats to sustainable agriculture and causing a decline in plant production by disrupting physiological, biochemical, and molecular functions. Salt stress disrupts germination, plant growth, photosynthesis, transpiration, and stomatal conductance [8,9]. As a result of soil salinity, the uptake of minerals by plants is disturbed due to the excessive accumulation of Na^+^ and Cl^−^ and the inhibition of K^+^ and Ca^2+^ uptake, which results in an ion imbalance [10,11]. Salt stress resulting from an excess of sodium ions (Na^+^) leads to a decrease in the content of chlorophyll and carotenoids, leaf necrosis, and a decrease in metabolic functions in the cell, including photosynthesis [12,13]. Salt stress is, therefore, a significant stress factor due to the combination of the effects of osmotic stress related to dehydration and damage related to the accumulation of Na^+^ ions, which can result in oxidative damage. As a result of the accumulation of Na^+^ ions, the ionic balance is upset, which reduces the water potential of the soil and leaves due to the difficulty of water uptake by plants. The effect is the disturbance of water relations and a decrease of turgor pressure in the plant, which leads to osmotic stress [9,14]. Salt stress due to Na^+^ accumulation induces premature aging, chlorosis, and necrosis of the leaves. These changes negatively affect protein synthesis and photosynthetic activity, which in turn leads to a lower proportion of carbohydrates in young leaves together with a reduction of the shoot growth rate [15]. As a result of the high concentration of salt in the soil environment, the photosynthetic apparatus is damaged and the stomata close, and the leaf expansion rate decreases [16]. Thus, salt stress-induced changes strongly affect the photosynthetic mechanism by affecting many essential proteins and produce changes in abundance related to redox state, phosphorylation, and synthesis/degradation [17]. Analysis of the fluorescence of chlorophyll a is an excellent tool for the quantitative determination of salt stress-induced damage to the photosynthetic apparatus, and photosystem II (PSII) damage can also be determined using this method [18,19,20]. Under the influence of salt stress, plants have developed several mechanisms at the cellular and tissue levels to avoid its effects. These mechanisms include changes in stomatal conductance, hormonal balance, the antioxidant defence system, osmotic regulation, and ion exclusion [21,22,23]. As a result, the leaf surface is reduced, thus limiting the photosynthetic process and, consequently, inhibiting plant growth. Salt stress, by limiting stomatal conductivity as a result of closing the stomata, leads to the inhibition of CO_2_ attachment and the stimulation of immense energy levels. This results in an increase in the level of reactive oxygen species (ROS) [23], which causes oxidative stress, as a result of overproduction and imbalance between defence mechanisms. The antioxidant system, which is classified as a defence mechanism, is composed of enzymatic antioxidants, which are: catalase (CAT), superoxide dismutase (SOD), ascorbate peroxidase (APX), monodehydroascorbate reductase (MDHAR), dehydroascorbate reductase (DHAR), glue -S-transferase (GST), etc. [24,25]. Rohman et al. [26] and Rohman et al. [27] showed that SOD activity was increased due to salt stress, suggesting an association between increased ROS production and a protective mechanism that reduces stress-induced oxidative damage. H_2_O_2_ is the product of SOD activity, and it is still reactive and dangerous to cells and must be eliminated by conversion to H_2_O in subsequent reactions. In plants, the enzymes regulating the cellular level of H_2_O_2_ are CAT and GPX [27,28,29]. SOD is therefore an enzyme that is the first line of defence against ROS, protecting the cell membrane system from damage [30]. CAT is a key enzyme that plays an important role in the cellular defence mechanisms against ROS. Its activity modulates the amounts of O_2_ and H_2_O_2_ and reduces the risk of the formation of OH radicals, which are highly reactive and can damage cell membranes, proteins, and DNA [28].

Plant cells also produce low molecular weight non-enzymatic antioxidants which are involved in the removal of ROS. These include phenolic compounds, which play an important role in maintaining the redox balance and improving the stress tolerance of plants. Therefore, the adaptation of plants to salt stress means the achievement of a balance between ROS and polyphenols and flavonoids [31]. These compounds play significant molecular and biochemical functions in plants, as signaling molecules in their defence and in mediating auxin transport. Phenolic compounds, including flavonoids, act as an antioxidant, scavenging free radicals in plants exposed to drought stress [32]. Flavonoids play a protective role under stressful conditions by neutralising radicals before they damage cells [33]. They are classified as essential secondary metabolites which are synthesised in almost every part of the plant in conditions of plant-environment communication.

One of the compounds classed as one of the flavonoids, which consist of three condensed rings and five hydroxyl groups, is quercetin (3, 5, 7, 3′, 4′-pentahydroxyflavone), a compound present in fruits, vegetables, wine, tea, etc. [34]. Metal ions, including Cu (II), can significantly change the chemical properties of quercetin, influencing its antioxidant and biological activity. The quercetin–copper complex (Q-Cu (II)) therefore shows higher antioxidant activity than pure quercetin. Copper is an essential metal for humans, animals, and plants, although it is also potentially toxic above optimal levels. In plants, it is an essential cofactor of many metalloproteins and is involved in several biochemical and physiological processes. Moreover, copper has a broad spectrum of effectiveness against pathogenic microorganisms, therefore it is also used as a plant protection agent in organic farming [35,36]. However, an excess of Cu induces oxidative stress inside plants through the increased production of ROS. Copper ions participating in initiating the process of H_2_O_2_ decomposition in the Fenton reaction are the first stage of inactivation of this ROS compound. Due to its dual nature (primary and potential toxicity), this metal encompasses a complex network of absorption, sequestration, transport, necessity, toxicity, and detoxification within plants. Therefore, it is important to monitor the biogeo-physicochemical behavior of Cu in the soil–plant–human systems, bearing in mind its possible essential and toxic roles [37].

Quercetin is a flavonoid of which various amounts are found in plants. There are numerous previous studies on the effects of inbuilt quercetin on physiological processes in plants. However, there is still little information on the effect of exogenous quercetin on the functioning of plants exposed to stress, including salt stress [38]. Previous studies on wheat seedlings sprayed with various concentrations of quercetin derivative showed the stimulating role of this flavonoid on physiological and biochemical processes [39], which allowed the use of this agent in studies on plants under salt stress. The aim of the research was to show the effect of the exogenous application of solutions of the Q-Cu (II), characterised by high antioxidant activity, on wheat seedlings growing under saline conditions, and in particular to assess the efficiency of the photosynthetic apparatus and its antioxidant properties.

## 2. Materials and Methods

### 2.1. Synthesis of the Quercetin—Copper (II) (Q-Cu (II)) Complex

The Q-Cu (II) complex was prepared according to the optimised method described by Bukhari et al. [36], slightly modified by increasing the amount of solvent used. Briefly, 0.001 mol of quercetin was dissolved in 300 mL of methanol. Then 0.002 moles of solid CuSO_4_ were added, and the solution was intensively stirred on a magnetic stirrer for 1.5 h. The characteristic yellow-brown solution was then filtered, concentrated, and dried using a vacuum evaporator (50 °C, 300 mbar).

### 2.2. Pot Experimental Design

The pot experiment was carried out at the University of Rzeszów (Poland). A soil with clay-sand grain size [40] and a slightly acidic reaction (KCl pH 6.35; H_2_O 6.52) was placed in plastic pots (11 × 11 cm, 3 kg soil/pot). The total contents of compounds in the soil were: phosphorus oxide (P_2_O_5_) 17.4 mg∙100 g^−1^, potassium oxide (K_2_O) 17.0 mg∙100 g^−1^, calcium (Ca) 9.46 mg∙100 g^−1^, and magnesium (Mg) 8.87 mg∙100 g^−1^. Four seeds of winter wheat of the Artist cultivar (breeder Deutsche Saatveredelung AG, Lippstadt, Germany) were put into each pot. The experiment was carried out in 4 replications in a growth chamber (Model GC-300/1000, JEIO Tech Co., Ltd., Seoul, South Korea) at a temperature of 22 ± 2 °C, humidity 60 ± 3% RH, photoperiod 16/8 h (L/D), and a maximum light intensity of about 300 µE m^−2^ s^−1^. During the experiment, the samples were kept at constant soil moisture of 50% of the maximum water holding capacity (WHC) in the pots. The pot setting was changed randomly every 7 days. When the plants reached the 14 BBCH phase (4 leaves unfolded) [41], salt stress was applied by watering the soil twice with 70 mL of 200 mM saline (NaCl) per pot, and at the same time, the control sample was watered with the same volume of deionised water. One day after the introduction of salt stress, the plants were sprayed with the Q-Cu (II) solution in the following concentrations: 100 mg∙L^−1^ (Q1), 500 mg∙L^−1^ (Q2), and 1000 mg∙L^−1^ (Q3). The quercetin derivative was diluted with ethanol (20 mL of solution for each pot). Spraying was performed twice 1 and 7 days after the application of the salt solution using a hand-sprayer. After physiological measurements were performed, the above-ground part of the plants was harvested for the determination of biochemical parameters (determination of the ROS level and enzyme activity).

### 2.3. Measurement of Physiological Parameters

Determination of the physiological parameters (chlorophyll content, chlorophyll fluorescence, and gas exchange) was carried out four times on the first fully developed wheat leaves: on the first and seventh days after each spraying.

During the experiment, the following measurements were carried out: relative chlorophyll content (CCI), and selected parameters of chlorophyll fluorescence (the maximum quantum yield of primary photochemistry [F_v_/F_0_], the photochemical efficiency of PS II [F_v_/F_m]_, and the performance index of PS II [PI]), the gas exchange (net photosynthetic rate [P_N]_, stomatal conductance [g_s_], transpiration rate [E] and intercellular CO_2_ concentration [Ci]).

#### 2.3.1. Relative Chlorophyll Content

Measurements were made using a hand-held CCM-200plus Chlorophyll Content Meter (Opti-Sciences, Hudson, NH, USA) calculating an index in CCI units based on absorbance at 650 and 940 nm. These measurements were made on full expanded wheat leaves. Five leaves per pot were analysed.

#### 2.3.2. Chlorophyll Fluorescence

Measurements of chlorophyll a fluorescence in leaves were performed with an apparatus (Pocket PEA, Hansatech Instruments, King’s Lynn, Norfolk, UK). The maximum available intensity was 3500 μmol which was applied for 1 s with light with a peak wavelength of 627 nm. The first fully developed leaves were dark-adapted for 30 min using leaf clips which were applied on adaxial leaf blades [42]. The following parameters were analysed during the test: the maximum quantum yield of primary photochemistry (F_v_/F_0_), the photochemical efficiency of PS II (F_v_/F_m_), and the performance index of PS II (PI). The chlorophyll *a* fluorescence parameters, including F_v_/F_m_, F_v_/F_0,_ and performance index (PI), were calculated with the programme Pocket PEA [43]. Two measurements of chlorophyll fluorescence were made per pot.

#### 2.3.3. Gas Exchange

A Portable Photosynthesis Measurement System LCpro-SD (ADC BioScientific Ltd., Hoddesdon, UK) was used to determine the gas exchange parameters: net photosynthetic rate (P_N_), transpiration rate (E), stomatal conductance (g_s_), and intercellular CO_2_ concentration (C_i_). During measurements, light intensity was 1300 µmol m^−2^ s^−1^ and the leaf chamber temperature was 22 °C. Two leaves were analysed from each pot.

### 2.4. Measurement of Biochemical Parameters

#### 2.4.1. Determination of the ROS Level

1 g of frozen tissue was homogenised with 4 mL of 50 mM chilled phosphate buffer (pH 7.4) to determine the level of reactive oxygen forms. The homogenate was then centrifuged at 10,000× *g* for 30 min (4 °C) and the supernatant thus obtained was collected for analysis. The ROS level in the extracts was determined by the fluorimetric method using 2′,7′-Dichlorodihydrofluorescein diacetate, according to the protocol described in Piechowiak and Balawejder [44]. The results were expressed as the increase in fluorescence/g of tissue/min.

#### 2.4.2. Determination of Enzyme Activity

One g of frozen tissue was homogenised with 4 mL of 0.9% NaCl solution containing 2% polyvinylpyrrolidone, 0.05% Triton X-100 and a mixture of protease inhibitors to determine the activity of SOD, CAT, and GPOX. The homogenates were then centrifuged at 10,000× *g* for 30 min (4 °C) and the supernatant obtained was then collected for analysis. The activity of superoxide dismutase was determined by the adrenaline method [45], the activity of catalase was determined by the method using ammonium metavanadate [46], and the activity of guaiacol peroxidase was determined according to the methodology described by Uarrot et al. [47]. The enzymatic activity in the extracts was standardised per mg of protein, the amount of which was determined by the Bradford method [48].

### 2.5. Statistical Analysis

The results were subjected to statistical analysis using Statistica 13.3.0 (TIBCO Software Inc., Palo Alto, CA, USA). Two-way ANOVA with repeated measurements (time evaluation as a factor) was used to verify the significance of the influence of soil salinity and spraying with the Q-Cu (II) solution on the physiological processes occurring in wheat seedlings. One-way ANOVA was used to determine the relationship for the determination of the ROS level and enzyme activity. Tukey’s HSD post-hoc test (*p* = 0.05) was used to determine the differences between the mean values of the parameters examined. Multiple regression analysis with stepwise selection of variables for the analysed parameters was calculated. The regression equation was given using the following equation:

$$Y=a+b_{1}\times X_{1}+b_{2}\times X_{2}+⃛+b_{p}\times X_{p}$$

## 3. Results

### 3.1. Relative Chlorophyll Content

**Figure 1:**
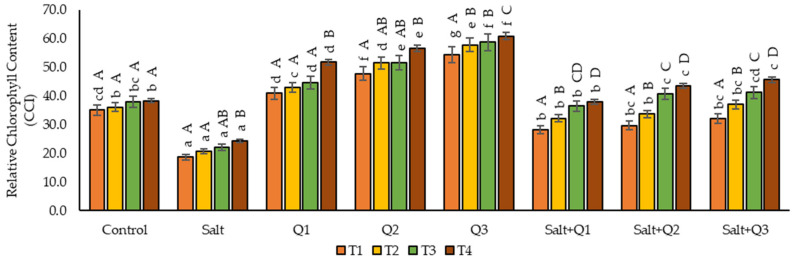
Effect of quercetin–copper complex (Q-Cu (II)) concentrations, salt stress, and terms of measurement on relative chlorophyll content (T1—the first day after the first application, T2—the seventh day after the first application, T3—the first day after the second application, T4—the seventh day after the second application). Capital letters indicate significant differences between the means at measurement terms for each variant of the experiment, lowercase letters indicate significant differences between the means at respective measurement term according to ANOVA followed by Tukey’s HSD test, *p* = 0.05).

Due to salt stress, the content of chlorophyll decreased compared to the control sample, especially in T1, when a decrease of as much as 87.2% was observed (Figure 1). As a result of the application of Q-Cu (II) to the plants not subjected to stress, a significant increase of the test parameter was found in proportion to the concentrations applied. The highest content of chlorophyll (60.8 CCI) was found in T4 as a result of using the highest concentration (Q3). In the case of plants subjected to salt stress, which was sprayed with Q-Cu (II), a significant increase in the chlorophyll content was found compared to plants that were not sprayed. However, their value did not differ from each other as a result of applying higher concentrations (Q2 and Q3). On the next measurement, dates increased content of chlorophyll was found in all variants of the experiment apart from the control sample, in which these values did not differ from each other on all measurement dates. The greatest increase in the content of chlorophyll was observed on the last measurement date (T4), which increased compared to the first measurement date (T1).

### 3.2. Chlorophyll Fluorescence

**Figure 2:**
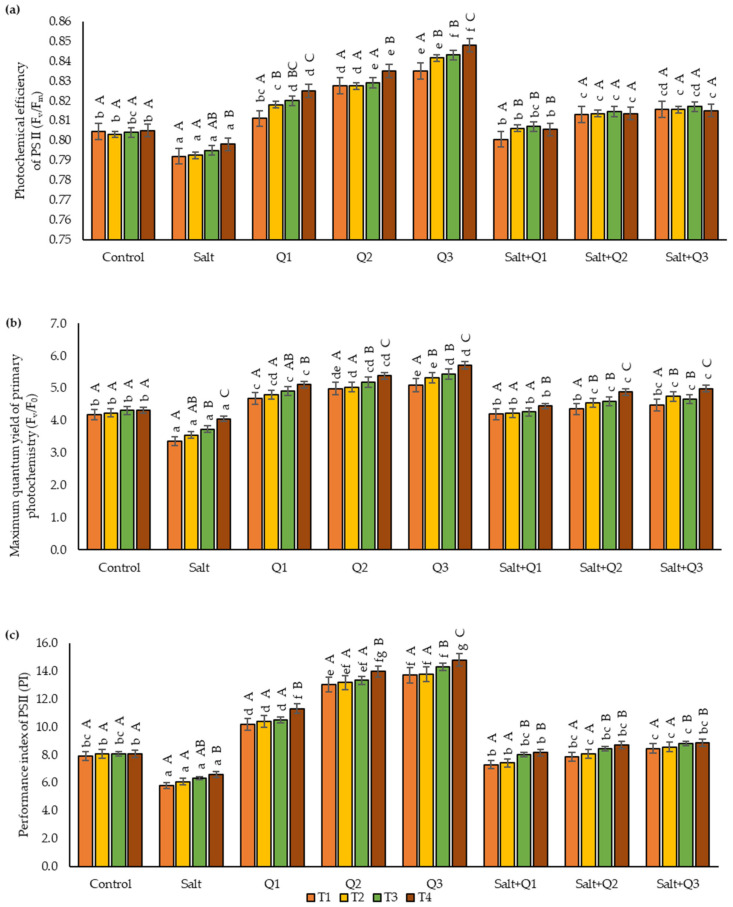
Effect of quercetin–copper complex (Q-Cu (II)) concentrations, salt stress, and terms of measurement on chlorophyll fluorescence parameters: (**a**) the photochemical efficiency of PS II (F_v_/F_m_); (**b**) the maximum quantum yield of primary photochemistry (F_v_/F_0_), and (**c**) the performance index of PS II (PI). (T1—the first day after the first application, T2—the seventh day after the first application, T3—the first day after the second application, T4—the seventh day after the second application). Capital letters indicate significant differences between the means at measurement terms for each variant of the experiment, lowercase letters indicate significant differences between the means at respective measurement term according to ANOVA followed by Tukey’s HSD test, *p* = 0.05).

For all the chlorophyll fluorescence parameters tested, a decrease in their value was observed as a result of salt stress compared to the control sample, only in T4 in the salinity variant, were there no significant changes in F_v_/F_0_ compared to the control sample (Figure 2a–c). Spraying wheat plants not subjected to salt stress with Q-Cu (II) solution caused an increase in the values of the chlorophyll fluorescence parameters that were examined in direct proportion to the concentrations used (the highest values of these parameters were found when Q3 was used). In the case of plants subjected to salt stress, spraying with Q-Cu (II) resulted in an improvement in the values of the parameters examined in comparison with plants growing in saline soil where no spraying was applied. However, in most cases, the use of higher concentrations did not cause significant increases in the values of the chlorophyll fluorescence parameters. The greatest difference in value was observed between Q1 and Q3. In the case of the F_v_/F_m_ and PI parameters, differences between Q1 and Q3 were observed in T1, T2, and T4, and in the case of F_v_/F_0_ in T2, T3, and T4. However, no differences between Q2 and Q3 were found in any of the parameters analysed. The values of the leaf chlorophyll fluorescence parameters that were tested increased with the sequential dates of the test. In all variants of the experiment, except for the control sample, an increase in the value of these parameters was observed with the passage of time.

### 3.3. Gas Exchange

**Figure 3:**
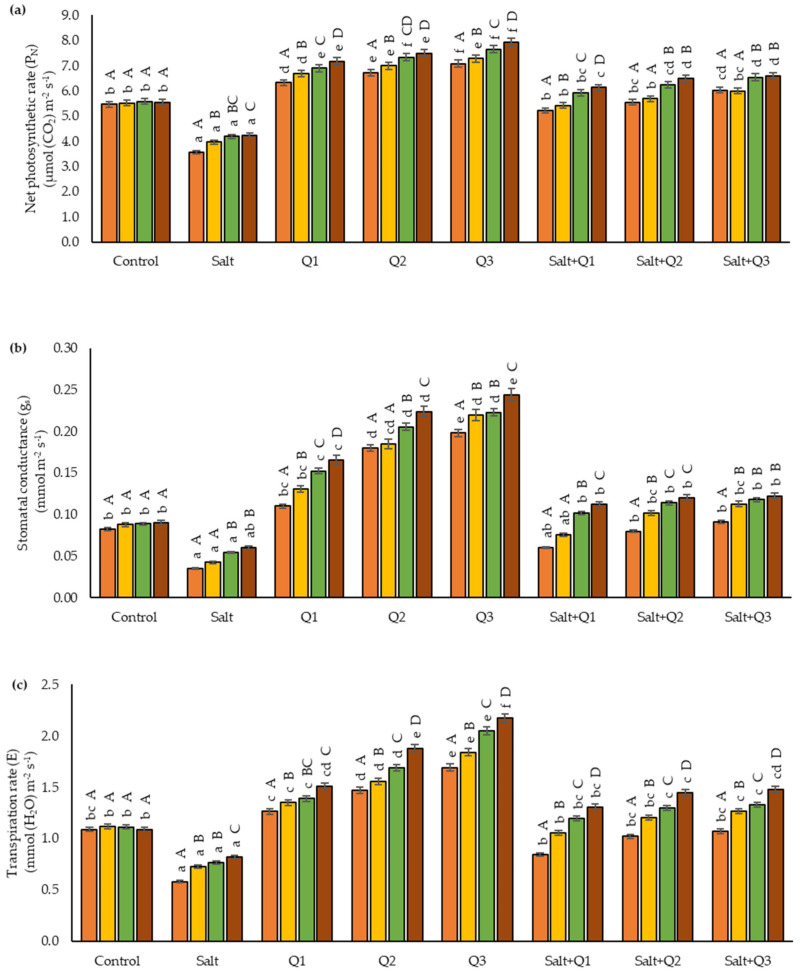

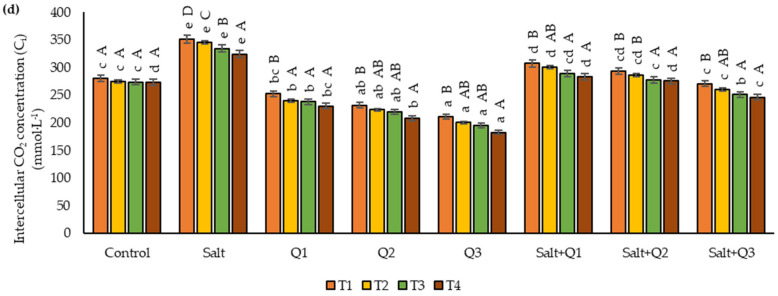
Effect of quercetin–copper complex (Q-Cu (II)) concentrations, salt stress, and terms of measurement on gas exchange parameters: (**a**) net photosynthetic rate (P_N_); (**b**) stomatal conductance (g_s_); (**c**) transpiration rate (E) and (**d**) intercellular CO_2_ concentration (C_i_). (T1—the first day after the first application, T2—the seventh day after the first application, T3—the first day after the second application, T4—the seventh day after the second application). Capital letters indicate significant differences between the means at measurement terms for each variant of the experiment, lowercase letters indicate significant differences between the means at respective measurement terms according to ANOVA followed by Tukey’s HSD test, *p* = 0.05).

Salt stress resulted in decreases of the parameter values (P_n_, g_s,_ and E), although in the case of gs in the last measurement period (T4)—(0.060 mmol∙m^−2^∙s^−1^), it did not differ significantly from the control sample (0.090 mmol∙m^−2^∙s^−1^) (Figure 3a–c). An increase in the value of most of the gas exchange parameters examined following the application of Q-Cu (II) solutions was observed in plants for which no salt stress was applied. The highest values for the P_n_, g_s,_ and E parameters were noted after spraying with concentration Q3. As a result of the application of Q-Cu (II) solutions to the seedlings subjected to salt stress, an increase in the value of these parameters was observed when compared to unsprayed plants, but only in the case of P_n_ (T1, T3, and T4) and E (T2) were significant differences found between the use of Q1 and Q3. In the case of the C_i_ parameter, the highest values were found in variants with saline soil, in particular in T1 and T2, where the value was higher by 25.7 and 25.8%, respectively, compared to the control sample (Figure 3d). As a result of the application of Q-Cu (II), a decrease in the value of C_i_ and an increase in the concentration of the quercetin derivative were observed. The lowest values of C_i_, by a significant margin, were noted when the concentration Q3 was used in plants growing on saline soil, in particular at T4 at which point the lowest value was obtained (183 mmol∙L^−1^). On saline soil, apart from the last measurement date (T4), the concentration Q3 did not significantly decrease C_i_ in comparison to Q2. The next measurement times showed an increase in the values of P_n_, gs, and E parameters, and a decrease in C_i_ were noted in all experimental variants except for the control sample.

### 3.4. Level of ROS

**Figure 4:**
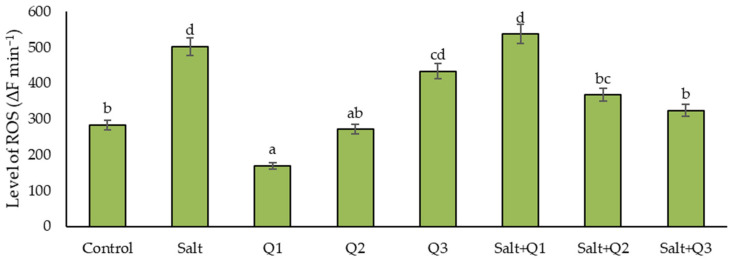
Effect of quercetin–copper complex (Q-Cu (II)) concentrations and salt stress on the level of reactive oxygen species (ROS). Different letters indicate significant differences between each variant of the experiment, according to ANOVA (followed by Tukey’s HSD test, *p* = 0.05).

The salinity of the soil caused a significant increase in the level of ROS compared to the control sample (Figure 4). When the Q1 (169.2 ∆F min^−1^) solution was sprayed on plants not subjected to salt stress, a decrease in the ROS level (by 67.3%) was found compared to the control sample with a gradual increase in cases where higher concentrations of Q-Cu (II) were used, in particular Q3, which reached a 53.2% higher value compared to the control sample. In plants subjected to salt stress, spraying with Q-Cu (II) solutions reduced the ROS level compared to the unsprayed variant, especially as a result of the use of the Q2 and Q3 concentrations (by 36.8 and 55.0%, respectively).

### 3.5. Activity of Enzymes

**Figure 5:**
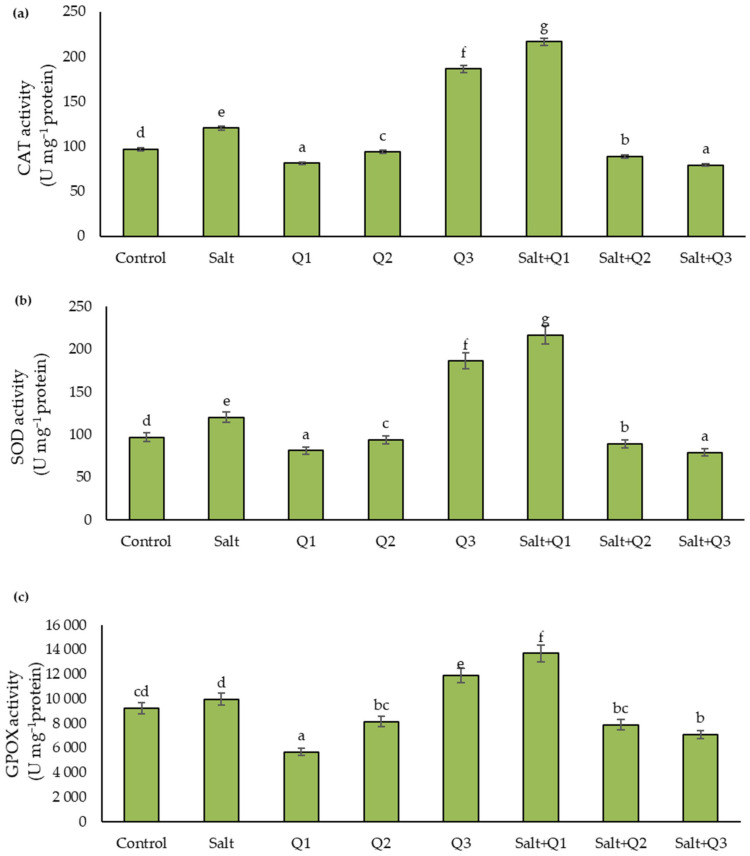
Effect of quercetin–copper complex (Q-Cu (II)) concentrations and salt stress on (**a**) catalase (CAT), (**b**) superoxide dismutase (SOD), and (**c**) guaiacol peroxidase (GPOX) activity. Different letters indicate significant differences between each variant of the experiment, according to ANOVA (followed by Tukey’s HSD test, *p* = 0.05).

The activity of the antioxidant enzymes tested increased as a result of salt stress when compared to the control sample, except for GPOX, where no significant changes were found (Figure 5a–c). Spraying with the Q1 solution caused a significant decrease in enzyme activity compared to the control sample, however, this activity increased after using the successively higher concentrations, in particular after using Q3 where for each enzyme tested, the activity was significantly higher compared to the control sample. In plants subjected to salt stress, which were sprayed with Q-Cu (II) solutions, the enzyme activity increased significantly, in particular as a result of the application of Q1. However, subsequent concentrations of Q-Cu (II) (Q2 and Q3) caused a significant decrease in the activity of all enzymes tested.

### 3.6. Regression Equation

To identify the independent variables influencing the physiological and biochemical parameters of plants, the multiple regression method was used (Table 1). The research shows a strong correlation between the tested physiological parameters and the concentration of the Q-Cu (II) complex used, the date of measurement, and salt stress. The models showed a good correlation with the explanatory variables. In the presented equations, the obtained values indicate the significance of the estimated regression parameters, and the equations are characterized by high coefficients of determination R^2^, which proves a very good fit to the model. In the case of Level of ROS, salt stress was included in the model, the fit was unsatisfactory (R^2^ = 0.325), and in the case of activity of enzymes, regression models could not be identified.

## 4. Discussion

### 4.1. Effect of Salt Stress on Wheat Plants

Photosynthesis is the process by which the energy needed to promote plant growth is generated. The components involved in the photosynthesis process, which include photosynthetic pigments, photosystems, and the enzymes involved in the carbon metabolism, are important in making this process effective and may be damaged as a result of salt stress [48]. In the present research, the influence of salt stress on disturbances in the functioning of physiological processes was demonstrated by unfavorable values of the parameters examined: chlorophyll content, chlorophyll fluorescence, and gas exchange.

The chlorophyll content in leaves was found to decrease as a result of salt stress. This can be explained as being due to the restriction of its accumulation as a result of the inhibition of several stages of porphyrin formation and the reduction of the production of chlorophyll-binding proteins [49]. In addition, salt stress can reduce photosynthetic pigments in chloroplasts, which reduces photosynthetic efficiency and final productivity [23]. 

In the experiment, a decrease in the values of all the chlorophyll fluorescence parameters analysed (F_v_/F_m_, F_v_/F_0,_ and PI) was noted in relation to the control samples. The value of the F_v_/F_m_ parameter under normal growth conditions is within the range of 0.780–0.840 and it is observed to decrease under salt stress [18]. In the present research, the value of this parameter in plants subjected to salt stress ranged from 0.756 (T1) to 0.772 (T4). The PI parameter is a very important and sensitive indicator of photosynthesis. Mehta et al. [19], in a study conducted on wheat seedlings growing under salt stress conditions, observed a decrease in the PI value compared to the control sample, which was also demonstrated in the present study. The decrease in the values of the chlorophyll fluorescence parameters and chlorophyll content can be explained by damage to the permeability of the cell membrane and disturbance of the functioning of thylakoids in chloroplasts, leading to a gradual decrease in the activity of photosystems [20,50]. The inhibition of physiological processes is related to the excessive accumulation of Na^+^ and Cl^−^ ions which causes a reduction in photosynthetic electron transport and photosynthetic efficiency [51], leading to significant inhibition of plant growth and, consequently, causing a lower grain yield. 

In a study by Saddiq et al. [6] and Kanwal et al. [52], in addition to the decrease in the parameters relating to chlorophyll fluorescence, the gas exchange parameters were also inhibited in plants subjected to salt stress. The excess of salt in the substrate causes the rapid closure of the stomata, leading to the inhibition of CO_2_ assimilation, which in turn disturbs the capture of light and use of energy, generating strong disturbances in the photosynthetic process [53]. The experiment showed a significant reduction in the values of the parameters (P_n_, g_s,_ and E) and an increase in C_i_ compared to the control sample. According to Guo et al. [54], wheat seedlings responded to salt stress with a reduction in the values of gas exchange parameters compared to normal conditions. Salt stress leads to the closure of the stomata and lower CO_2_ assimilation and a reduced rate of transpiration. This can be explained by a decrease in the activity of the Rubisco enzyme, which converts CO_2_ into high-energy substances. This is due to the cleavage of the large Rubisco subunit by ROS under stress conditions [55]. ROS are generated as a result of stress, including salt stress, which can cause the degradation of proteins (including membrane linking proteins, chlorophyll proteins) necessary for the hooking of phycobilisomes on to thylakoids [56]. In addition, ROS bursting causes damage to the thylakoid membrane, leading to modulation in membrane protein profiles, a decrease in the activity of the oxygen-evolving complex of PSII, and an increase in PSI activity [49].

### 4.2. Effect of Exogenous Application of Q-Cu (II) on Wheat Plants

#### 4.2.1. Effect on Plants Not Exposed to Salt Stress

As a result of the stress factors to which crops are exposed, it is reasonable to use substances—phytoprotectants—that protect plants by alleviating the impacts of their effects [57]. These metabolites include, in particular, phenolic compounds produced by the shikimate-phenylpropanoid biosynthetic pathway, which include quercetin [26]. In previous studies conducted by Jańczak-Pieniążek et al. [39] and Migut et al. [58], as a result of spraying with quercetin derivative (potassium quercetin complex), a positive, proportional to the concentrations used, effect of this flavonoid was demonstrated. This resulted in an improvement in the values of content of chlorophyll and selected parameters of chlorophyll fluorescence and gas exchange. In the presented studies on the effect of the Q-Cu (II) complex on wheat plants not exposed to salt stress, its positive effect on the content of chlorophyll was also found. The value of this parameter increased significantly, which was proportional to the concentrations used, and reached the highest value as a result of the application of the Q3 concentration, which was recorded at each measurement date. The stimulating effect of exogenous application of quercetin was also found by Parvin et al. [57], who in the studies on tomato seedlings obtained a higher content of chlorophyll compared to the control sample. As a result of Q-Cu (II) spraying on plants not exposed to salt stress, an improvement in the photosynthesis process was also found, which was confirmed by an improvement in the values of chlorophyll fluorescence parameters and gas exchange. According to Dobrikova and Apostolova [59], the application of quercetin may change the photosynthetic properties caused by structural changes in the thylakoid membranes, leading to an increased degree of thylakoid light-scattering dispersion. This may affect the transfer of electrons between the pigment–protein complex, which leads to an increase in the transfer from PSII to PSI, improving the photosynthesis process. Also, the research conducted by Yildiztugay et al. [60] showed a positive effect of the exogenous application of the flavonoid—naringenin on the photosynthesis process. In these studies, after the application of flavonoid, as in our studies, an increase in the values of chlorophyll fluorescence and gas exchange parameters was found compared to the control, which contributed to the improvement of the photosynthesis process. 

#### 4.2.2. Effect on Plants Exposed to Salt Stress

The use of a quercetin derivative that ensures tolerance to biotic and abiotic stresses is even more justified due to the limitations of the biological potential of varieties [38,61,62]. Quercetin is a flavonoid that plays a significant role in maintaining a balanced concentration of ROS in cells and enhancing physiological functions in order to tolerate environmental stress [38]. The application of quercetin having the -OH group at the 3-position of the flavonoid skeleton makes it an effective scavenger of ROS, inhibiting their aggregation [63,64]. The presence of metal ions has a great influence on the biological activity of quercetin. The hydroxy and oxy group in quercetin has the ability to form complexes with metal ions. Bukhari et al. [36] showed that the quercetin–copper complex has a higher antioxidant activity than pure quercetin, which suggests that Cu (II) ions significantly change its chemical properties. Also, Pękal et al. [65] showed that the Q-Cu (II) complex has stronger antioxidant activity, which suggests that the complex has greater radical scavenging activity than free quercetin. Quercetin prevents limiting the degree of stomatal closure by reducing the level of H_2_O_2_ which contributes to their closure [63,64]. H_2_O_2_, a form of ROS, is a secondary messenger in the ABA-signaling network and is believed to be necessary for the closure of the stomata. ABA regulates the opening and closing of the stomata, while the presence of quercetin in cells disrupts the normal signaling pathway. Thus, the use of exogenous quercetin affects the obstruction of stomatal closure by inhibiting mitogen-activated protein kinase (MAPK) activity related to ABA signaling [38,66]. The antagonistic effect of quercetin on ABA-mediated closure of the stomata may not only result from limiting the accumulation of H_2_O_2_, but also from suppressing the activity of MAPKs that act on H_2_O_2_ to trigger the movement of the stomata [67]. This dependence was also confirmed in our research in which, as a result of spraying wheat plants growing on saline soil with Q-Cu (II) solutions, stomatal conductivity (g_s_) increased, which resulted in an improvement in other gas exchange parameters. The application of quercetin also resulted in an increase in the parameter values of chlorophyll fluorescence and chlorophyll content. The protective effect of the exogenous application of quercetin on the photosynthetic apparatus was also confirmed in the studies conducted by Dobrikova and Apostolova [59] in which plants were exposed to UV radiation. This proves that quercetin induces structural changes in thylakoid membranes, which is one possible cause of the protection of the photosynthetic apparatus. The antioxidant effect of quercetin was also confirmed in studies by Kurep et al. [68], in which different plant species were stressed by paraquat, proving that quercetin is an effective protective agent against the harmful effects of ROS on plants. Therefore, this flavonoid has a beneficial effect on the tolerance of plants to oxidative stress caused by the interaction of ROS with chloroplast electron transport. The prevention of ROS formation by flavonoids is the result of inhibition of the action of ROS-producing enzymes, including lipoxygenase (LOX), monooxygenase (MO), and xanthine oxidase (XO), chelating metal ions (Fe, Cu) [69]. The present studies have confirmed the strong antioxidant activity of the Q-Cu (II) complex. Exposure of wheat to salt stress resulted in an increase in ROS production, which corresponded to a higher activity of CAT and SOD (in the case of GPOX, the increase in activity was statistically insignificant). A similar relationship was shown in studies by Rohman et al. [27] in which a higher level of ROS was found in plants subjected to salt stress than in control samples. The application of the Q-Cu (II) complex at concentrations Q2 and Q3 abolished the oxidative stress caused by salt stress. The level of ROS, as well as the activity of SOD and GPOX, was lower in these variants than in the variants subjected to salt stress without the application of Q-Cu (II). Moreover, Q-Cu (II) at concentration Q1 decreased the ROS level in the samples not exposed to salt stress, and this corresponded to lower CAT, SOD, and GPOX activity than in the control sample. This activity was probably connected to the antioxidant properties of the Q-Cu (II) complex, which is well described by Bukhari et al. [36]. The highest concentration of Q-Cu (II) (Q3) showed a pro-oxidative effect, which resulted in a rapid increase in the production of ROS and the activity of all the antioxidant enzymes examined. Parvin et al. [57], in studies on tomato seedlings growing under salt stress conditions to which quercetin was applied, also showed a similar relationship. The addition of exogenous quercetin to saline-treated plants resulted in a decrease in the activity of antioxidant enzymes compared to the variant without the application of this flavonoid. This may indicate that there is a lower production of ROS as a result of the action of quercetin, which has strong scavenging properties. This in turn causes a decrease in the activity of antioxidant enzymes.

The presented research on wheat plants shows that the Q-Cu (II) complex has valuable antioxidant properties. This may contribute to the improvement of the course of physiological and biochemical processes in cultivated plants and counteract the effects of salt stress.

## 5. Conclusions

The research conducted has shown that stress-related to soil salinity has a negative impact on the photosynthetic process, as evidenced by the deterioration of the parameters examined. The foliar application of Q-Cu (II) solutions improved the values of these parameters in plants not subjected to salt stress in direct proportion to the concentrations of Q-Cu (II) applied. When plants in the variant subjected to salt stress were sprayed, an improvement in the values of physiological parameters was found. In most of the cases analysed, however, no difference was found between the application of the concentrations Q2 and Q3. Salt stress increased the level of ROS and the activity of the antioxidant enzymes. Spraying with Q-Cu (II) solutions caused, in the case of the concentrations Q2 and Q3, a decrease in the ROS level and the activity of the enzymes that were tested, which proves the strong antioxidant role of this flavonoid derivative in scavenging the reactive ROS. As a result of the research conducted, it was found that the concentrations 500 mg∙L^−1^ (Q2) and 1000 mg∙L^−1^ (Q3) have the most beneficial effect on the protection of plants against salt stress, which may help to counteract and alleviate the effects of this stress in the future, as well as contributing to the implementation of sustainable agricultural practices. In the future, it is planned to conduct further studies in which the characterization of metal complex osmolytes measurement—oxidative stress parameter will be performed, including malondialdehyde (MDA) and hydrogen peroxide (H_2_O_2_) level. Moreover, it is planned to verify the obtained results in field conditions.

## Figures and Tables

**Table 1:** Regression equations for physiological and biochemical processes in wheat plants.

Parameters		Regression Equation	R^2^
	CCL	y = 31.007 ***−15.164 salt stress *** + 6.284 concentration *** + 2.980 term ***	0.939
Chlorophyll Fluorescence	F_v_/F_m_	y = 0.804 *** + 0.010 concentration ***−0.016 salt stress *** + 0.002 term ***	0.919
	F_v_/F_0_	y = 4.031 *** + 0.356 concentration ***−0.600 salt stress *** + 0.141 term ***	0.931
	PI	y = 8.702 ***−3.825 salt stress *** + 1.455 concentration *** + 0.260 term	0.885
Gas Exchange	P_N_	y = 5.101 *** + 0.674 concentration ***−1.244 salt stress *** + 0.247 term ***	0.886
	g_s_	y= 0.080 ***+ 0.033 concentration ***−0.074 salt stress ***+ 0.013 term ***	0.892
	E	y = 0.899 *** + 0.231 concentration ***−0.429 salt stress *** + 0.109 term ***	0.918
	C_i_	y = 289.875 *** + 60.125 salt stress ***−25.563 concentration ***−7.188 term ***	0.977
	ROS	y = 289.134 *** + 143.379 salt stress **	0.325
Activity of Enzymes	CAT	A regression model has not been identified	
	SOD	A regression model has not been identified	
	GPOX	A regression model has not been identified	

Significant at: ** *p* < 0.01, *** *p* < 0.001.

## Data Availability

Not applicable.
